# Impaired Cell Cycle Regulation in a Natural Equine Model of Asthma

**DOI:** 10.1371/journal.pone.0136103

**Published:** 2015-08-20

**Authors:** Alicja Pacholewska, Vidhya Jagannathan, Michaela Drögemüller, Jolanta Klukowska-Rötzler, Simone Lanz, Eman Hamza, Emmanouil T. Dermitzakis, Eliane Marti, Tosso Leeb, Vincent Gerber

**Affiliations:** 1 Swiss Institute of Equine Medicine, Vetsuisse Faculty, University of Bern and Agroscope, Bern, Switzerland; 2 Institute of Genetics, Vetsuisse Faculty, University of Bern, Bern, Switzerland; 3 Division of Pediatric Hematology/Oncology, Department of Pediatrics, Bern University Hospital, Bern, Switzerland; 4 Clinical Immunology Group, Department of Clinical Research and Veterinary Public Health, University of Bern, Bern, Switzerland; 5 Department of Zoonoses, Faculty of Veterinary Medicine, Cairo University, Cairo, Egypt; 6 Department of Genetic Medicine and Development, University of Geneva Medical School, Geneva, Switzerland; 7 Institute of Genetics and Genomics in Geneva, Swiss Institute of Bioinformatics, Geneva, Switzerland; University of Sydney, AUSTRALIA

## Abstract

Recurrent airway obstruction (RAO) is a common and potentially debilitating lower airway disease in horses, which shares many similarities with human asthma. In susceptible horses RAO exacerbation is caused by environmental allergens and irritants present in hay dust. The objective of this study was the identification of genes and pathways involved in the pathology of RAO by global transcriptome analyses in stimulated peripheral blood mononuclear cells (PBMCs). We performed RNA-seq on PBMCs derived from 40 RAO affected and 45 control horses belonging to three cohorts of Warmblood horses: two half-sib families and one group of unrelated horses. PBMCs were stimulated with hay dust extract, lipopolysaccharides, a recombinant parasite antigen, or left unstimulated. The total dataset consisted of 561 individual samples. We detected significant differences in the expression profiles between RAO and control horses. Differential expression (DE) was most marked upon stimulation with hay dust extract. An important novel finding was a strong upregulation of *CXCL13* together with many genes involved in cell cycle regulation in stimulated samples from RAO affected horses, in addition to changes in the expression of several HIF-1 transcription factor target genes. The RAO condition alters systemic changes observed as differential expression profiles of PBMCs. Those changes also depended on the cohort and stimulation of the samples and were dominated by genes involved in immune cell trafficking, development, and cell cycle regulation. Our findings indicate an important role of CXCL13, likely macrophage or Th17 derived, and the cell cycle regulator CDC20 in the immune response in RAO.

## Introduction

According to the World Allergy Organization 300 million people suffer from asthma. Asthma is a public health problem worldwide and its prevalence is still increasing [1]. There are several types of asthma described, which vary regarding their pathogenesis, molecular mechanisms and clinical phenotype. Specifically, cytokine expression profiles and the types of T helper cells implicated in the immune response after allergenic stimulation show considerable variation, as reviewed in [2].

Asthma has an estimated heritability of more than 60% [3,4], but in accordance with the considerable immunological and phenotypical variation, it is genetically heterogenous [5] and more than 200 genes have been shown to be related to asthma [6]. Furthermore, there are also important environmental risk factors, including indoor and outdoor allergens, such as mites or pollens, and irritants like lipopolysaccharides (LPS).

Animal, predominantly murine, models are extensively studied to improve our understanding of the complex immunogenetic background of asthma [6]. However, murine models have important limitations, since mice have a short life span and do not spontaneously develop asthma, but must undergo sensitization protocols with allergen challenges [7]. Apart from humans, allergic asthmatic conditions occur naturally only in cats and horses [8]. The asthma-like disease in horses is called recurrent airway obstruction (RAO), which is a debilitating and incurable respiratory disease affecting stabled mature horses worldwide [9–11]. RAO-affected horses develop marked airway obstruction due to bronchospasm, inflammation, mucus accumulation and remodelling resulting in severe clinical signs such as coughing, respiratory distress and increased breathing effort during periods of exacerbation, which are triggered by hypersensitivity reactions to allergens and irritants mainly from hay dust [11–14]. Affected horses may be treated medically or by allergen avoidance and can live longer than 25 years. These characteristics make RAO a unique animal model for human asthma [11,15].

While the immunogenetic background of RAO is still not completely understood, several studies have shown that interactions of innate and adaptive immune responses play an important role [11]. Importantly, we have previously shown strong genetic effects. The prevalence of RAO is 3-5-fold increased among offspring from affected stallions compared to controls, and the heritability is high with a complex mode of inheritance involving several major genes suggesting genetic heterogeneity of RAO [14,16,17]. Moreover, we have demonstrated an association of RAO in horses with an augmented resistance against intestinal strongylids manifested by decreased shedding of parasite eggs [10,13,18]. These studies showed that RAO-affected horses had a significantly lower likelihood of egg shedding compared to unaffected horses. However, this association appears to depend on the genetic background with a marked relationship between RAO and strongylid egg shedding in one, but not another high-prevalence family.

Gene expression studies may potentially highlight genes that play a key role in the response to RAO-related antigens. Some studies showed contradictory results regarding the involvement of cytokines characteristic for Th1 or Th2 type immune response and it has been suggested that cytokine profiles reflecting both types of Th responses are observed at different time-points after antigen challenge [9]. In addition, an increased expression level of *IL17*, which is characteristic for Th17 cells, was shown to be associated with RAO [19]. However, only limited sets of specific candidate genes have been investigated in RAO so far [15,18,20–24].

In this study, we therefore chose a more comprehensive approach and compared global gene expression levels between RAO-affected and control horses. We hypothesized that antigen challenge in RAO-affected horses has not only local effects in the lungs but can also elicit systemic responses that would be reflected in the gene expression profiles of peripheral blood mononuclear cells (PBMCs). We were particularly interested in the differences in gene expression upon stimulation with hay dust extract (HDE) as the main causative environmental factor in RAO. HDE is a mixture of molecules (e.g. mold spores, inorganic dust, plant fragments) [25] and it contains LPS [26,27]. We also performed cell stimulations with LPS alone to be able to distinguish between LPS-mediated and allergen-specific effects of HDE. In addition, we analysed differences in the immune response with respect to strongylid parasite antigen (recombinant cyathostomin antigen, RCA) in RAO compared to non-affected horses. Finally, we investigated the influence of the host genetics on RAO by studying the expression profiles of three cohorts of horses with different genetic backgrounds.

## Material and Methods

### Ethics Statement

All animal experiments were performed according to the local regulations and with the consent of the horse owners. This study was approved by the Animal Experimentation Committee of the Canton of Bern, Switzerland (BE33/07, BE58/10 and BE10/13). The sample collection was previously described in detail in an earlier publication [18].

### Horses and Phenotyping

A total of 85 animals were selected from three cohorts of Warmblood horses: two half-sibling families, consisting of the descendants of two RAO-affected sires (Family 1, Family 2) and one group of unrelated horses (Unrelated). These animals were already used in earlier studies [18,28]. Family 1 consisted of 9 RAO-affected (RAO) and 8 control (CTL) horses. Family 2 consisted of 8 RAO and 9 CTL, and Unrelated of 23 RAO and 28 CTL horses. Horses in Family 1 and Family 2 were paternal half-siblings with different mothers. Based on pedigree records comprising 7 ancestral generations, the horses in the families had inbreeding levels of less than 1%, calculated with MTDFREML [29]. The group of unrelated horses consisted of a subset of horses described in Kehrli et al [30]. The horses did not share parents and, as far as feasible based on the available pedigree information, were also unrelated at the grandparent level. The RAO phenotype (RAO/CTL) was assigned according to the Horse Owner Assessed Respiratory Signs Index (HOARSI), as described previously by Ramseyer et al. [31] and validated by Laumen et al. [32]: Control horses had HOARSI 1, defined by the absence of coughing or nasal discharge episodes; all RAO-affected horses had HOARSI 3 or 4, showing moderate to severe clinical signs of respiratory disease. Case definition for HOARSI 3 is abnormal breathing, regular or frequent coughing, or both. HOARSI 4 is defined by poor performance in addition to the same clinical signs as HOARSI 3. The intermediate milder phenotype of HOARSI 2 was not included in this study. The classification refers to the period when the horses were exhibiting their most severe clinical signs. At the time of the study, the RAO-affected horses were kept in a “low dust” environment and were in complete or partial remission showing either no or only mild clinical signs (mainly mildly increased breathing effort). All horses underwent a thorough clinical examination to exclude any systemic or localized infectious diseases. None of the horses had received any medication for treatment of RAO or any anthelmintic treatment within at least three months preceding the examination and blood collection to avoid any effects on the responses to stimulations.

### Samples, Sequencing, and Data Preparation

The blood sample collection, isolation of peripheral blood mononuclear cells, and RNA purification were performed as described in Lanz et al. [18]. We used the RNA TruSeq Sample Preparation Kit v2 for library preparation (guide Part #15026495 Rev.D, Illumina) and sequenced the libraries on the Illumina HiSeq2000 platform using 2 x 50 bp paired-end sequencing cycles. For this study we used RNA-seq data derived from PBMCs stimulated for 24 h with LPS (250 ng/ml), HDE (12, 9, or 6 μg/ml), RCA (4 or 1 μg/ml), or unstimulated (mock) as described in Pacholewska et al. [28]. Some of the cell stimulations or RNA extractions failed. Therefore, the RNA-seq data were generated from the 561 available RNA libraries out of the full set of 595 samples (94%). The exact number of biological replicates per group is shown in Table 1.

**Table 1:** Number of biological replicates per group studied. The matrix shows the number of biological replicates in each group (Mock–unstimulated cells, LPS–lipopolysaccharides, HDE–hay dust extract, RCA–recombinant cyathostomin antigen).

Stimulating agent^a^	Family 1		Family 2		Unrelated	
	RAO	Control	RAO	Control	RAO	Control
Mock	6	7	6	9	23	27
LPS	8	8	6	9	23	27
RCA_1	8	7	6	9	23	27
RCA_4	9	7	6	9	23	27
HDE_6	8	8	6	9	23	27
HDE_9	9	7	6	9	23	27
HDE_12	8	8	6	9	23	27

^a^The numbers indicate the concentration in μg/ml.

We prepared non-stranded libraries and collected 17 million read pairs per library on average. The reads mapped to the horse genome reference (EquCab2, [33]) with high efficiency (more than 90% mapped reads). All 561 binary alignment files (BAMs) were used previously for an assembly of the equine PBMC transcriptome [28] and are available from the European Nucleotide Archive database (http://www.ebi.ac.uk/ena/data/view/PRJEB7497). Reads uniquely mapped to annotated transcripts (Ensembl, release 72) were counted gene-wise with the HTSeq software [34]. More details on sequence quality control and mapping can be found in Pacholewska et al. [28].

### Data Quality Control

In order to look for an overall experiment effect or any batch effect on the clustering of the samples, we performed a principal component analysis. The raw counts were normalized using variance-stabilizing transformation included in the DESeq2 R package [35]. The 500 most variable genes were used for this analysis. The samples were then plotted across the first three principal components.

Based on multidimensional scaling (MDS) plots the decision of merging different concentrations of the same agent was made, as explained in the Results section (S1 Fig).

### DE Analysis

We used the R package edgeR for the analyses of DE, since this package offered the most comprehensive generalized linear model, which was essential for the analysis of our multifactorial experiment [36]. The R source codes are available in S1 File.

Genes with an expression level less than 1 cpm at least in the mean number of samples per group (8 for Family 1 and Family 2; 25 for Unrelated) were excluded from further analysis. Raw counts were then normalized with trimmed means of M values included in the edgeR package. The data were fitted using the generalized linear model (GLM), which allows for multiple comparisons using only one design model, and tested for DE.

Based on Fisher’s exact test we observed significant differences in the number of differentially expressed (DE) genes (false discovery rate threshold: FDR < 0.05) among the families and therefore cohort was taken as factor influencing the gene expression level in the experimental design (S2 Fig).

For the comparison of the RAO effect in the different horse cohorts upon each of the stimulations, we used a model with coefficients for every group studied. In total, we had 24 groups: 2 conditions (RAO, control) * 4 stimuli (mock, LPS, HDE, RCA) * 3 cohorts (Family 1, Family 2, Unrelated). The gene-wise negative binomial generalized linear model was used for fitting the data. The fitted data were then tested for differential expression between RAO and control horses using likelihood ratio tests and appropriate contrasts (S1 File). To determine genes differently regulated in RAO samples, relative to control samples, upon at least one of the stimulations in at least one of the cohorts we applied a statistical model with coefficiencies for RAO status, stimulation, and cohort with interactions (S1 File).

DE genes were then used for pathway analysis with the GeneGo software (MetaCore by Thomson Reuters) and GeneCodis (Gene Ontology (GO) Annotations Human (EBI), version 106) [37–40] using the orthologus human genes annotations. Human gene symbols were assigned to the Ensembl equine gene IDs using Ensembl Biomart (version 72) [41]. In GeneGo the probability for a random intersection of an input list with a set IDs in ontology groups is calculated as using the hypergeometric distribution p-value.

## Results

### Experimental Design & Data Collection

For a global transcriptome analysis we isolated PBMCs from RAO-affected (RAO) and control (CTL) horses (2 conditions) belonging to two different half-sib families or a cohort of unrelated horses (3 cohorts). PBMCs were stimulated with HDE, LPS, RCA or left unstimulated (4 treatments). In total, the material consisted of 561 RNA samples derived from 85 horses. More details on the sample numbers and design of the analysis are given in Table 1.

### Data Quality Assessment

**Fig 1:**
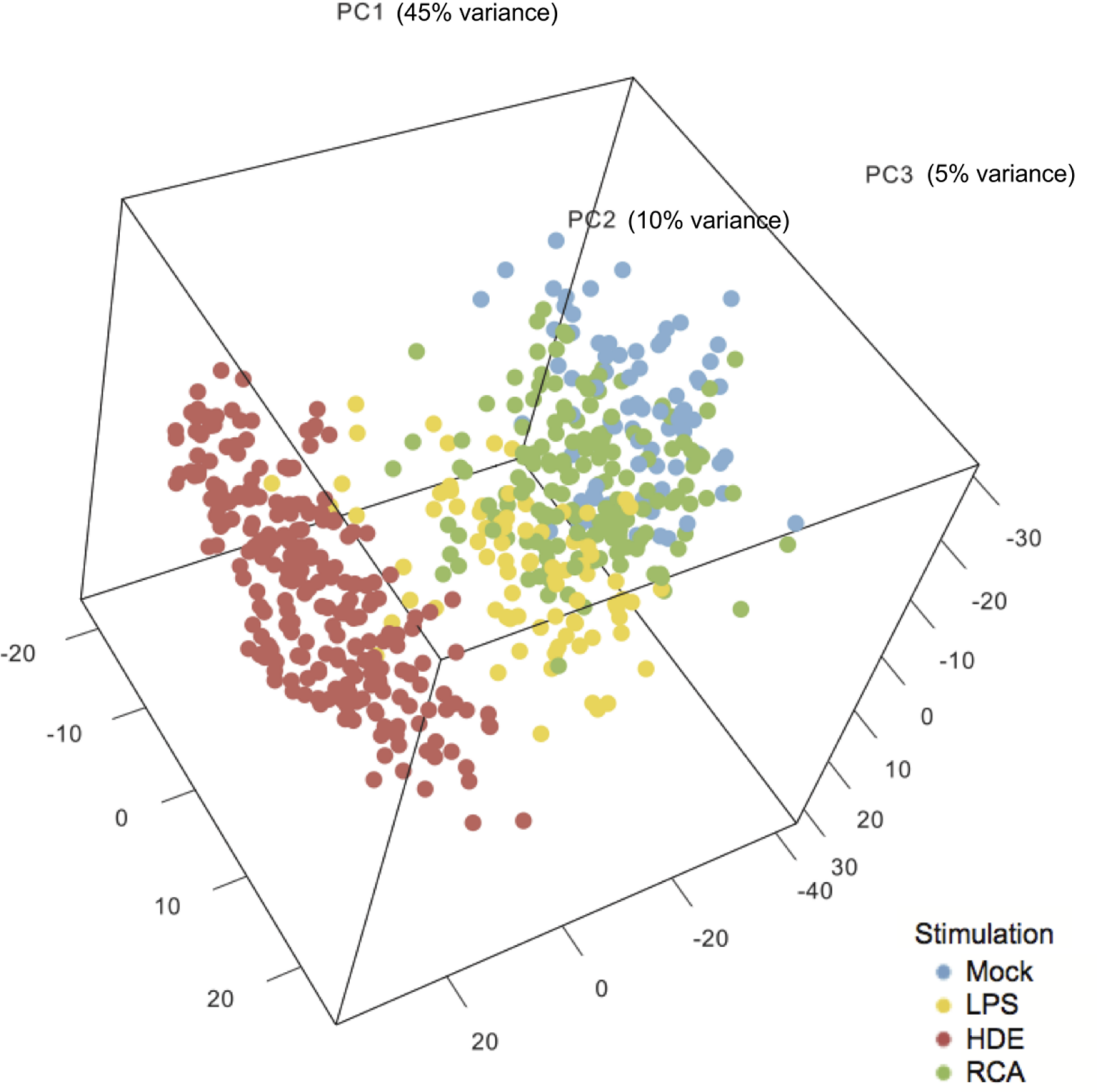
Quality control of the RNA-seq data. The figure shows a principal component analysis of the individual samples plotted across the three most variable components. The circles on the MDS plot represent the individual samples and are colored according to the 4 different stimulations: no stimulating factor (mock), lipopolysaccharides (LPS), recombinant cyathostomin antigen (RCA, both concentrations), and hay dust extract (HDE, all three concentrations).

The MDS plot showed clear separation between the stimulation groups and concordance among group replicates. The analysis showed that RCA-stimulated samples were the least distant and the HDE-stimulated samples were the most distant samples from the unstimulated samples (Fig 1). Although the HDE-stimulated samples were less distant from the LPS-stimulated samples, they created a separate cluster, indicating that HDE-specific factors affect the gene expression profile of PBMCs.

The MDS analysis revealed that samples derived from the same horse, but stimulated with different concentrations of the same agent, tightly clustered together (S1 Fig). Therefore, different concentrations of the same agent were merged for the subsequent analysis and treated as a single stimulation. This resulted in more robust DE analysis with more samples used.

Of the 26,991 equine genes annotated in Ensembl, 13,265 had more than 1 cpm (read count per million reads) at least in the mean number of samples per group to be included in the DE analysis (S1 File).

### Evaluation of Stimulation with LPS

LPS is a potent stimulator of innate immunity and has been studied in detail in several cells and immune related tissues [42–44]. As an additional quality control experiment we compared the expression profiles of LPS-stimulated and unstimulated PBMCs from control horses. The identified 3,787 statistically significantly DE genes (FDR < 0.05) consisted of several cytokines in addition to previously identified LPS response genes like *IL1B*, *IL6*, *IL8*, *IL15*, *IFNG* and chemokines like *CCL22*, *CXCL2*, *CXCL6* [42]. Also the DE genes consisted of LPS upregulated protein kinases like *STAT3* and cell division proteins like *CD80*, *CD86* as shown in Deifl et al. [45].

In addition, we subjected the DE genes with HGNC symbols to enrichment analysis using GenoGo Metacore. The most significantly enriched GeneGo pathway was the naive CD4+ T cell differentiation (FDR = 6.92e-6), which includes the highly upregulated *IL6* (log 2 fold change = 2.90). The expression of *IL6* is known to be induced by LPS through the nuclear factor κβ (NF-κB) transcription factor [46]. GeneGo provides a list of GO processes significant for a given pathway map. Nine out of the ten most significant pathways enriched for DE genes showed significance for the GO process cellular response to LPS (GO:0071222). These results were in agreement with our expectations and confirmed successful stimulation of cells with LPS.

### RAO-status Effect

**Fig 2:**
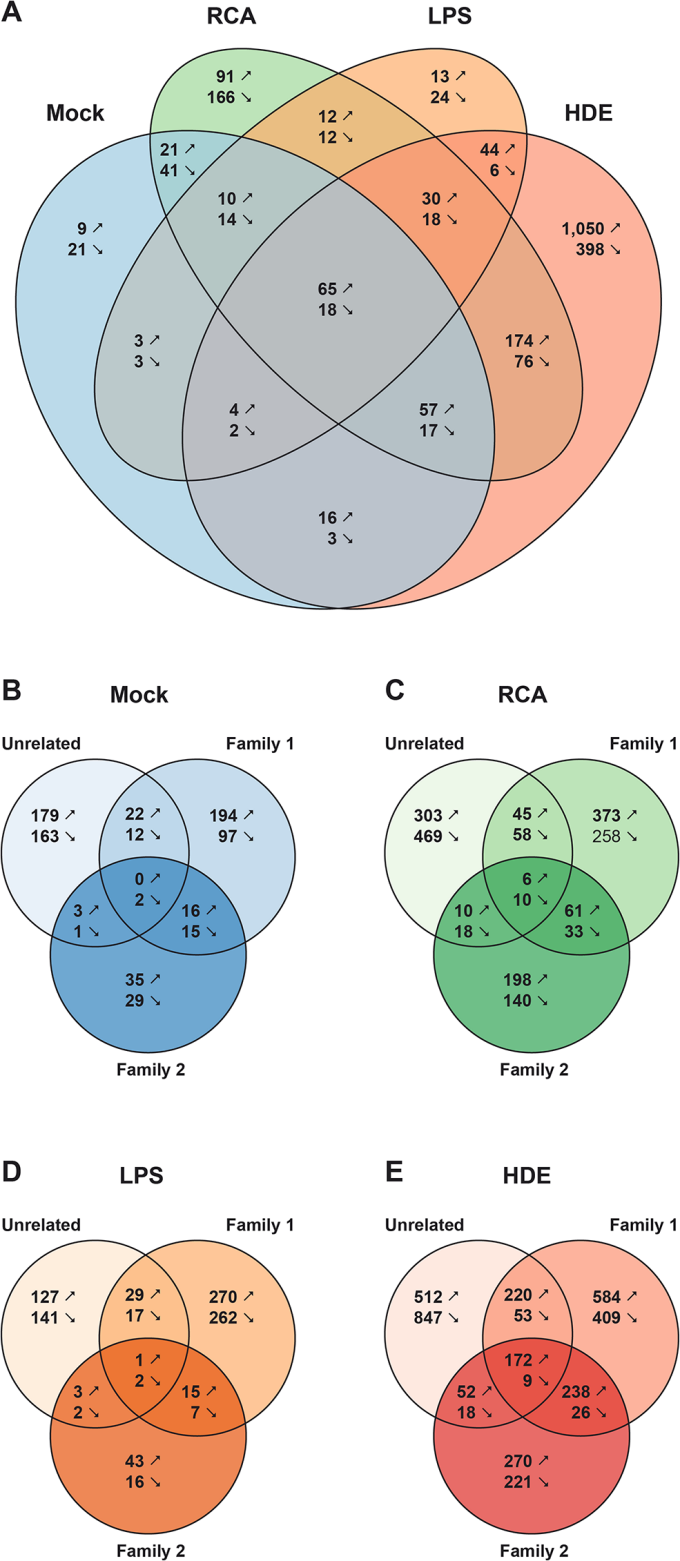
Venn diagrams illustrating the number of DE genes between RAO and control horses. Panel A illustrates the number of DE genes upon each stimulation across all horses. The panels B-E further differentiate this dependent on the three different cohorts. (B) Unstimulated cells (mock), followed by cells stimulated with (C) recombinant cyathostomin antigen (RCA), (D) lipopolysaccharides (LPS), and (E) hay dust extract (HDE). The numbers represent the number of significantly up (↗) and down (↘) regulated genes in RAO samples compared to control samples.

After all quality control experiments had yielded satisfactory results, we started to address the differences between RAO-affected and control horses. Pairwise comparisons revealed the greatest difference in the gene expression in RAO samples upon stimulation with HDE. Stimulation with LPS revealed a similar number of RAO-regulated DE genes as in the unstimulated cells (Fig 2A). In Family 2 the number of DE genes was smaller than in the other cohorts (Fig 2B–2E). The whole list of DE genes and the detailed results of the gene enrichment analysis are given in S1, S2 and S3 Tables.

The GeneGo tool “compare experiments workflow” intersects DE gene lists in terms of their mapping to gene ontologies and pathway maps. Hence this tool can be used to study differential pathway enrichment in different DE gene lists. This tool subclassifies the overlapping gene lists as “common” set for all genes found in each of the lists, “similar” set comprising all partial intersections and a “unique” set for every list.

**Fig 3:**
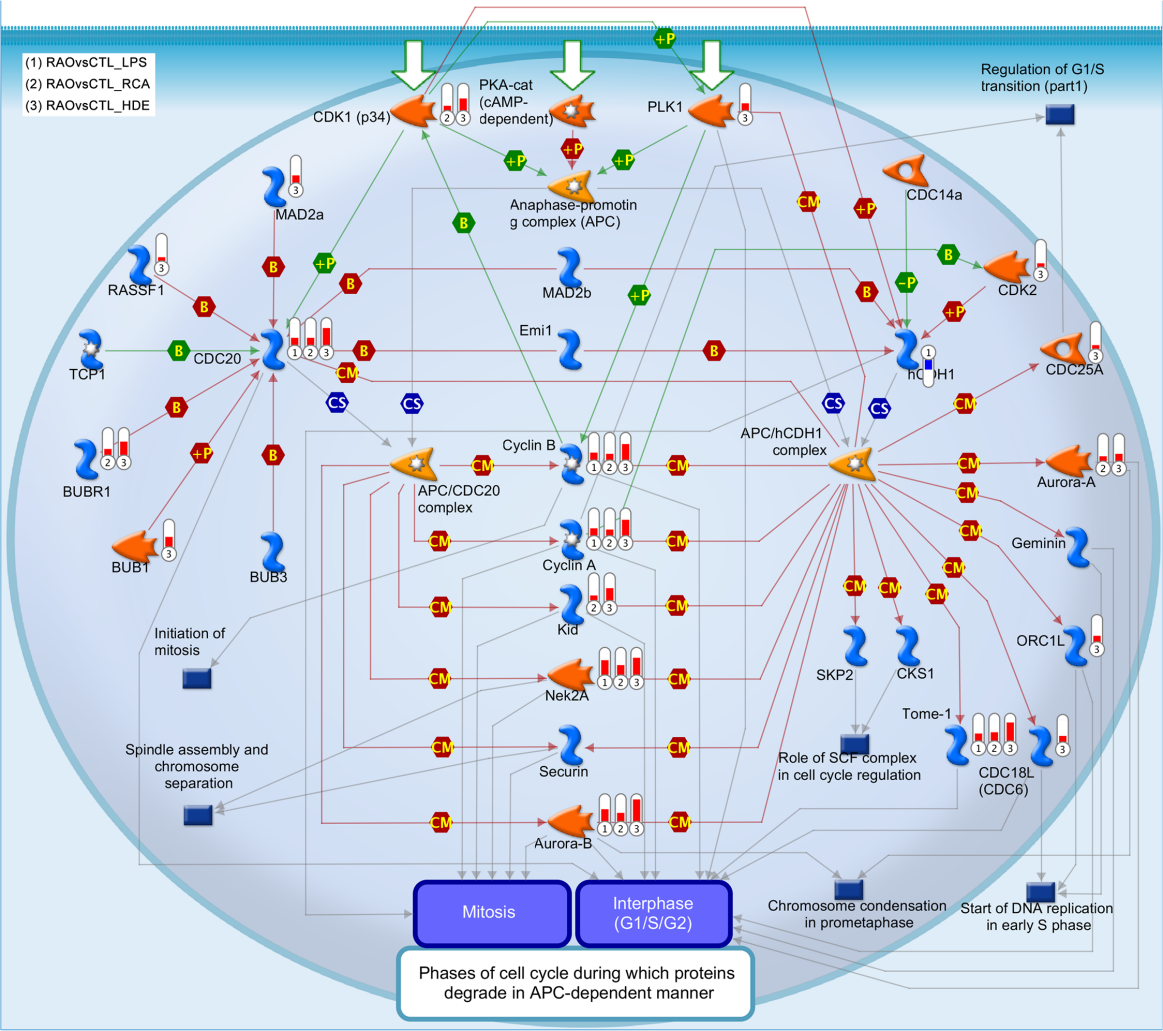
GeneGo pathway “role of anaphase-promoting complex (APC) in cell cycle regulation”. The thermometers indicate the object regulating genes were up- (red) or downregulated (blue) by the RAO condition upon stimulation with 1) lipopolysaccharides (LPS), 2) recombinant cyathostomin antigen (RCA), and 3) hay dust extract (HDE). Lines indicate activation (green), inhibition (red) or unspecified (grey) interactions between the molecules. The object shapes correspond to molecule type and are described in S3 Fig and at https://portal.genego.com/legends/MetaCoreQuickReferenceGuide.pdf.

Ontology enrichment and canonical pathway analysis for common genes suggested several functions and pathways involved mainly in cell cycle regulation and immune response (S3 Table). Interesting to note in pathway analysis for LPS/HDE/RCA stimulation was “role of anaphase-promoting complex (APC) in cell cycle regulation” (Fig 3).

While the pairwise comparisons revealed almost 3,900 DE genes between RAO and control samples upon at least one of the stimulations and cohorts (Fig 2, S1 Table), the overall RAO effect regulated 3,508 genes (S2 Table). This overall RAO-effect represents differences in gene expression in RAO compared to control samples, regardless of stimulation or cohort. The GeneGo map folder consists of pathways grouped into regulatory, metabolic, disease, toxicity and drug action sections. Enrichment analysis with map folders with differentially expressed genes regulated by overall RAO effect showed the ‘asthma’ folder as the top statistically significant folder with the regulation of epithelial-to-mesenchymal transition and extracellular matrix remodelling as top enriched pathways.

### Additional factors altering RAO-regulated genes

The gene regulation by RAO depended on the stimulation agent and/or cohort that further modified the gene ‘response’ with respect to the RAO baseline effect, i.e. in unstimulated cells in the Unrelated group of horses (Table 2).

**Table 2:** Genes regulated by RAO-status with further dependencies on stimulating agent and/or cohort. For every effect the number of DE genes and the top ten DE genes are listed. The overall “conditionRAO” effect includes significant genes due to the RAO effect in at least one cohort and stimulation. Mock-stimulated samples from control horses of the Unrelated cohort were used as a reference group. Baseline “conditionRAO” effect represents differences between RAO and control samples from the Unrelated unstimulated group. Colons “:” indicate an interaction effect, e.g. “conditionRAO:Family1” effect represents differential changes in the RAO effect in Family 1 in mock compared to RAO effect in Unrelated group in mock.

Effect	# DE genes	Top ten genes
conditionRAO–overall	3,508	*GPNMB*, *MMP3*, *ENSECAG00000003774*, *SERPINH1*, *CXCL13*, *CD163L1*, *IFNG*, *APOBEC3Z1B*, *NDUFA4L2*, *MMP-1*
conditionRAO–baseline (Unrelated, mock)	382	*VNN1*, *CCL24*, *GPT2*, *MMP1*, *FER1L6*, *SLIT3*, *APOBEC3Z1B*, *CCL26*, *COL4A1*, *SLC22A3*, *UHRF1*
conditionRAO:cohortFamily1	357	*MMP3*, *CXCL17*, *ENSECAG00000001949*, *ENSECAG00000003099*, *GPT2*, *MARCO*, *PPP1R36*, *TG*, *CCL24*, *APOBEC3Z1B*
conditionRAO:cohortFamily2	175	*MMP3*, *SELP*, *TGFB3*, *FBP1*, *VNN1*, *CCL24*, *APOBEC3Z1B*, *CCL26*, *ENSECAG00000023871*, *ENSECAG00000024588*
conditionRAO:stimulusHDE	428	*MMP3*, *EREG*, *CXCL13*, *MARCO*, *CXCL6*, *CD163L1*, *IFNG*, *NDUFA4L2*, *MMP-1*, *CCL13*
conditionRAO:stimulusHDE:cohortFamily1	140	*GPR1*, *EREG*, *STAB1*, *MRC1*, *MMP19*, *CXCL13*, *MARCO*, *TFPI2*, *CCL24*, *CCL13*
conditionRAO:stimulusHDE:cohortFamily2	97	*MMP3*, *ENSECAG00000001910*, *EREG*, *NTRK1*, *IFNG*, *ENTPD3*, *FBP1*, *CCL26*, *ENSECAG00000023871*, *CCL13*
conditionRAO:stimulusLPS	68	*MMP3*, *PTX3*, *SERPINH1*, *CXCL6*, *ALDH1A2*, *FBP1*, *CCL24*, *MMP-1*, *RNase_MRP*, *5_8S_rRNA*
conditionRAO:stimulusLPS:cohortFamily1	20	*G-CSF*, *MMP19*, *MARCO*, *TREM2*, *IL8*, *ENSECAG00000015885*, *FBP1*, *CCL24*, *F3*, *CLEC4F*
conditionRAO:stimulusLPS:cohortFamily2	21	*MMP3*, *PTX3*, *SERPINH1*, *NTRK1*, *ALDH1A2*, *TGFB3*, *ENTPD3*, *FBP1*, *CCL13*, *ENSECAG00000024588*
conditionRAO:stimulusRCA	61	*MMP3*, *PDE11A*, *MARCO*, *CXCL6*, *SPP1*, *LAMA4*, *MMP12*, *FABP4*, *SEPP1*, *RN18S*
conditionRAO:stimulusRCA:cohortFamily1	15	*MMP3*, *MARCO*, *SLC47A1*, *CXCL6*, *ENSECAG00000013872*, *ENSECAG00000015885*, *SPP1*, *MMP12*, *FABP4*, *ENSECAG00000023871*
conditionRAO:stimulusRCA:cohortFamily2	14	*MMP3*, *ENSECAG00000003801*, *SERPINH1*, *SLC27A6*, *CD5L*, *AMPP*, *SEPP1*, *RN18S*, *RN18S*, *ENSECAG00000027634*

**Fig 4:**
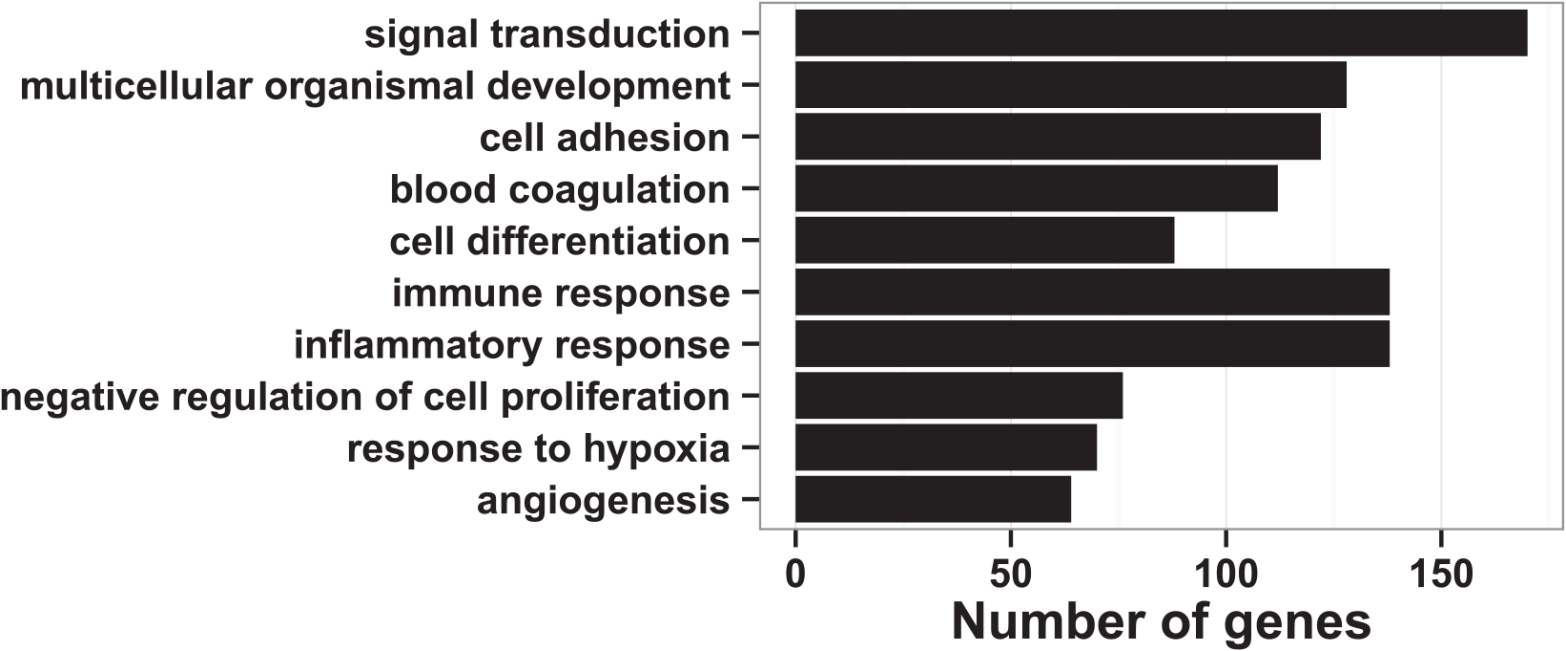
Biological processes enriched by RAO-regulated genes. The bars represent the number of RAO-regulated genes by the overall RAO-effect, regardless stimulation and/or cohort.

The RAO regulated genes comprised of genes involved in e.g. immune response, cell differentiation, and response to hypoxia as shown in Fig 4.

## Discussion

In this study, we investigated the influence of different antigenic and irritant stimuli on the gene expression profiles of PBMCs from genetically distinct groups of horses affected by RAO, an asthma-like condition. In contrast to the rodent models typically used in asthma research, equine RAO is a wide-spread naturally occurring disorder. The results of our study show that stimulation with HDE strongly affects the gene expression profile of equine PBMCs. The observed RAO-dependent effects of allergenic and irritant stimuli on PBMCs support the hypothesis that this asthma-like disease causes a systemic immune response. HDE, which is believed to be the major trigger of RAO, induced differential gene expression in a much broader set of genes than stimulation with LPS, which is one of the components of hay dust and is also known to provoke a strong immune response. Genes that are affected by RAO status (regardless of the specific stimulation) may be of importance for our understanding of the pathomechanisms involved in RAO.

We also observed the impact of the RAO condition in response to parasite antigen with almost 300 DE genes altered by both HDE and RCA, providing further evidence that RAO and other equine hypersensitivity disorders like urticarial reactions and multiple hypersensitivities [10,13,30] are associated with altered defence against parasites.

In addition, we also observed differences in the number of DE genes between RAO and CTL samples between the cohorts, which support our hypothesis that differences in the genetic background of RAO affect the immunological response to specific stimuli (Fig 2B–2E). Family 1 and the Unrelated horses showed more genes affected by RCA compared to Family 2. Interestingly, this is in accordance with earlier results demonstrating an association of RAO with increased parasite resistance in Family 1 and unrelated RAO-affected horses, but not in Family 2 based on strongylid egg counts in faecal samples [10,13].

One of the top genes altered by the conditionRAO:cohortFamily1 effect was *CCL24* that is located in a genomic region previously identified to be associated with RAO in this family [14]. This gene was also differentially expressed in Family 2, however, the effect was smaller than in Family 1. We did not observe significant changes in the expression of the *SOCS5* gene that has been associated with RAO condition in Family 2.

These results further support the hypothesis of genetic heterogeneity in RAO, which was previously postulated based on genetic and gene expression data [14,18,20]. The effect of the genetic background on RAO needs further investigation, preferably, including genomic data.

**Fig 5:**
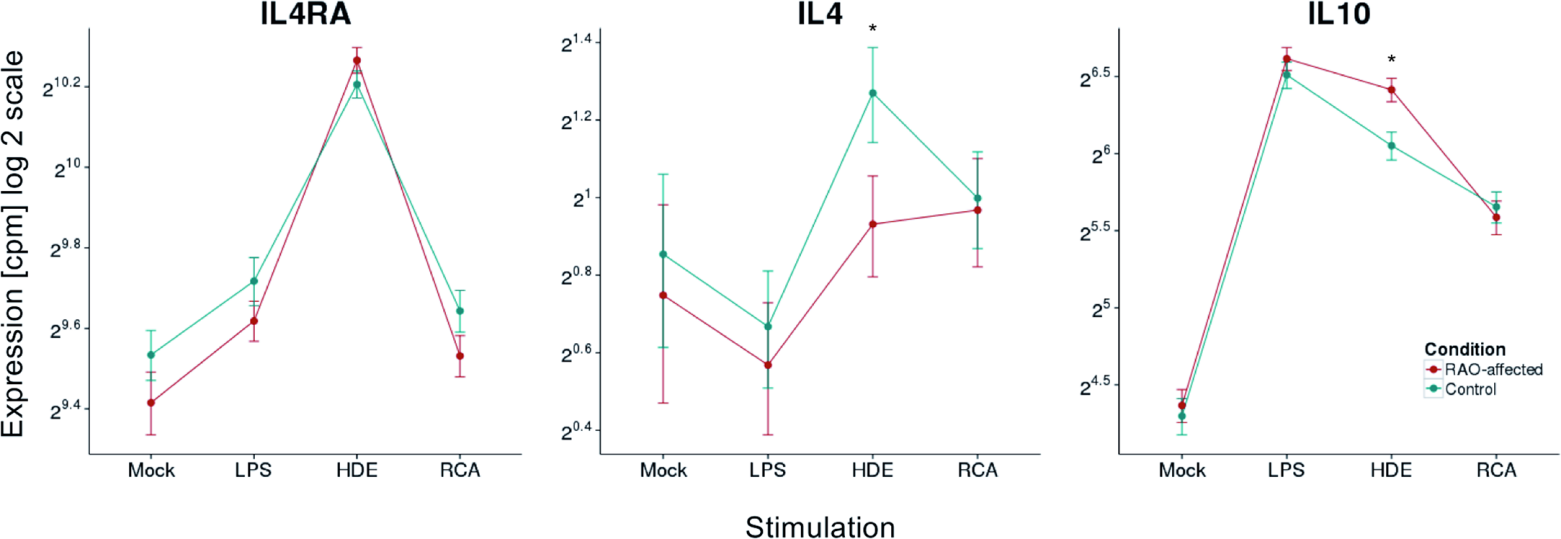
Expression level of cytokines previously identified as RAO-upregulated. The graphs represents the expression of *IL4R*, *IL4*, and *IL10* that were identified to have significantly increased expression levels in RAO horses in Family 1 by qRT-PCR [18]. The x-axes represent the stimulations: no stimulating factor (mock), lipopolysaccharides (LPS), recombinant cyathostomin antigen (RCA), and hay dust extract (HDE). On the logarithmic scaled (log 2) y-axes mean expression values are given in normalized counts per million. Error bars represent 95% confidence intervals. The stars (*) represent significant changes between expression levels of RAO (red dots and lines) and control (green dots and lines) samples at false discovery rate < 0.05.

A previous expression study showed an increased expression of *IL4*, *IL4R*, and *IL10* upon stimulation with HDE in RAO compared to non-affected horses [18]. We could confirm the upregulation of *IL10* in our data. *IL4R* was also upregulated in RAO samples, with stronger upregulation in Family 1 compared to Family 2, albeit below the significance threshold (Fig 5). In contrast, the RAO-regulation of *IL4* in contrast to the previous study showed a differential expression in the opposite direction. The *IL4* gene had overall low expression levels across the samples and DE analysis of RNA-seq data has been shown to present problems with lowly expressed genes [47–49]. Moreover, the previously published qRT-PCR expression study measured *IL4* gene expression in comparison to 18S rRNA as a standard. In the present data rRNA was removed during poly(A)-selection in our library preparation and hence we could not investigate this aspect in detail.

**Fig 6:**
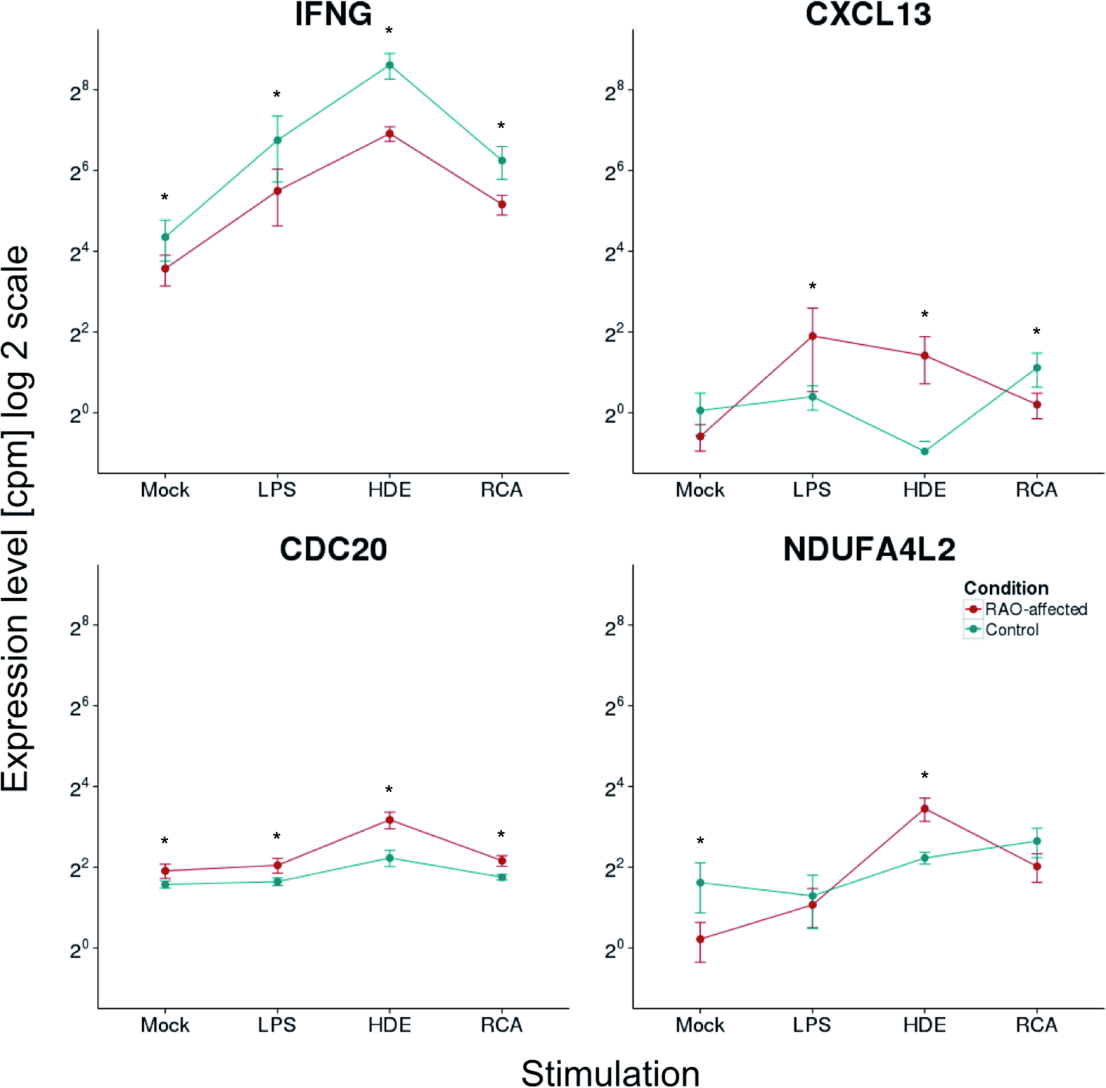
Expression of RAO-relevant genes. Expression levels of *IFNG*, *CXCL13*, *CDC20*, and *NDUFA4L2* are shown. The x-axes represent the stimulations: no stimulating factor (mock), lipopolysaccharides (LPS), recombinant cyathostomin antigen (RCA), and hay dust extract (HDE). On the logarithmic scaled (log 2) y-axes mean expression values are given in normalized counts per million. Error bars represent 95% confidence intervals. The stars (*) represent significant changes between expression levels of RAO (red dots and lines) and control (green dots and lines) samples at false discovery rate < 0.05.

The overall RAO effect showed 3,508 genes to be up- or downregulated regardless of stimulation or cohort. These genes were mostly enriched for pathways related to human asthma supporting the idea of RAO in horses being a good natural model for studies on asthma. Interestingly, the pathway “role of APC in cell cycle regulation” has not been listed as significant asthma-related pathway in the GeneGo collection yet, as only one object of this pathway (*RASSF1*) has been described to be associated with asthma [50]. Our data, however, showed that this pathway is significantly related to RAO-response with many objects upregulated, especially upon stimulation with the RAO-related antigen HDE (Fig 3). APC can be activated by one of two activators: cell division cycle 20 (CDC20) or its homolog, cadherin 1 type 1 (CDH1). These two factors act at different stages of the cell cycle to regulate distinct functions. CDC20 is active from metaphase through anaphase and promotes separation of sister chromatids, whereas CDH1 acts from the end of mitosis and prevents premature entry into S phase, as reviewed in [51–53]. The upregulated *CDC20* in RAO samples may thus suggest increased cell proliferation in RAO-samples (Fig 6).

The most RAO-downregulated gene upon stimulation with HDE was the interferon gamma gene (*IFNG*). Interferon gamma is crucial for the function of both innate and adaptive immune responses e.g. due to its stimulatory function on macrophages [54–56]. Indeed, we observed both types of immune response to be affected by the RAO condition, and therefore the decrease in *IFNG* in RAO horses could be involved in the RAO pathomechanism (Fig 6).

### Innate Response

The HDE stimulation resulted in a significant upregulation of genes involved in the innate immune response, e.g. complement system proteins (*C1S*, *C1R*, *C1QC*, *C2*) and complement component receptors (*C3AR1*, *C5AR2*) in the samples of RAO-affected compared to control horses. The role of the complement system in asthma has previously been described with the main focus on C3 and C5 complexes [57–61].

We also observed increased levels of *IL34* mRNA in RAO samples treated with LPS or HDE. This cytokine supports the viability of monocytes and promotes the formation of macrophages [62]. It is crucial for the differentiation and maintenance of Langerhans cells and tissue resident macrophages and with its receptor, colony-stimulating factor-1 receptor, represents a novel target for the treatment of several chronic inflammatory conditions [63,64].

In addition, the upregulated chemokines (*CXCL12*, *CXCL14*, *CCL14*) also attract and/or activate monocytes [65–68]. *CXCL13* is known as a B-cell attractant in germinal centres [69,70], but monocytes and macrophages are the major source of *CXCL13* [70]. This chemokine was the most significantly upregulated DE gene when RAO samples stimulated with HDE were compared to controls stimulated with HDE (Fig 6, S1 Table). Moreover, it was upregulated following stimulation with LPS, downregulated upon RCA stimulation, and no significant changes were observed in unstimulated cells (Fig 6, S1 Table). Secretion of CXCL13 protein seems to occur only after cell activation, and circulating monocytes have been shown to express *CXCL13* when stimulated by LPS [70].

### Th1/Th2 Types of Adaptive Response

Our data did not indicate any bias towards either a Th1 or a Th2 type response. Previous studies investigating the type of response in RAO have found conflicting results. The time point of sampling and the duration of stimulation, respectively, may influence results [9]. In the present study, we observed downregulation of both Th1 type (*IFNG*) and Th2-type cytokines (*IL4*, *IL13*) in RAO cells stimulated with HDE. This may be explained by increased levels of the interleukin *IL10* in the HDE-stimulated RAO samples, which is known to inhibit the expression of Th1- and Th2-type cytokines [71] and has previously been reported to be upregulated in RAO [18,72]. Interestingly, increased *IL10* is associated with decreased *IL4* expression in parasitized horses [73].

Although it was shown that CXCL13 is not produced by either Th1 nor Th2 cells, it is produced by human Th17 cells [74]. The Th17 cell subset is known for its role in autoimmune and inflammatory diseases [58,75]. In asthmatic patients the number of Th17 cells in blood and BALF is increased, positively correlating with asthma severity and airway remodelling [76,77]. Since our study was performed on cell mixtures, we cannot rule out the possibility that the increase in *CXCL13* might be due to an expansion of Th17 cells within the PBMCs rather than the above mentioned monocyte activation.

### Response to Hypoxia

The overall RAO effect revealed a significant role of hypoxia inducible genes with enrichment of 55 from 175 genes annotated in GO:0001666. We found a mitochondrial NADH dehydrogenase 1 alpha subcomplex 4-like 2 gene (*NDUFA4L2*) downregulated in unstimulated RAO PBMCs, but upregulated upon stimulation with HDE (Fig 6). *NDUFA4L2* is a target gene for hypoxia-inducible factor 1 (HIF-1) [78]. Little is known about the physiological function of the NDUFA4L2 protein, but it limits reactive oxygen species production in cells under hypoxic conditions [78]. The expression of the HIF-1 alpha subunit gene (*HIF1A*) was shown to be upregulated in lung cells of horses with RAO. The *HIF1A* expression level was correlated with the severity of the disease [79] in agreement with the higher expression of HIF-1 in asthmatic bronchial biopsies after antigen challenge [80].

From 392 human HIF-1 target genes identified by [81–83] and obtained via the PAZAR database [84] we identified a substantial proportion (n = 53; 14%) with significantly different expression levels between RAO and CTL in our data. Most of these DE genes (n = 32; 60%) showed an RAO-dependent expression difference only upon stimulation with HDE (S1 Table).

Hypoxia occurs during bronchial asthma attacks [85–87], but a hypoxic microenvironment is characteristic for inflamed tissues in general [88,89]. Furthermore, it has been shown that HIF-1 potentiates allergic inflammation in airways even without true hypoxia [90–92]. During a hypoxic response, the HIF-1α molecule is stabilized and assembles with HIF-1β to form the HIF-1 complex. The activated HIF-1 acts on the hypoxia responsible elements (HRE) in the promoters of target genes and regulates their expression [93]. The HIF-1α subunit is also stabilized in a hypoxia-independent manner that involves proteins of the NF-κB, TNF-α, or TGFβ1 signalling cascades [94–96]. Moreover, HIF-1α expression was also shown to be increased in a time- and dose-dependent manner by LPS in a macrophage-derived cell line [97]. In our study, each stimulating agent investigated (LPS, RCA and HDE) significantly increased (in comparison to unstimulated cells) the expression level of the *HIF1A* gene and *NFKB2* encoding a subunit of the NF-κB transcription factor, which activates cellular signalling pathways via dynamic modulation of cytokines, chemokines and other signalling molecules, and is an important signalling element of the immune response in asthma [98]. However, the RAO condition did not show any significant impact on the *HIF1A* or *NFKB2* expression.

The importance of the HIF-1 signalling pathway in the asthmatic airways lies partially in the fact that it activates many genes implicated in tissue remodelling. Airway remodelling is a characteristic feature of asthma and it involves increased mucus production, fibrosis, thickening of epithelial and smooth muscle layers, and angiogenesis [99]. Many of these processes are also observed in airway remodelling of equine RAO [11]. The resulting obstructed and hyperresponsive airways disrupt the proper functioning of the lungs [99].

### Anti-apoptosis in RAO

We speculate that the differential *CXCL13* expression is unlikely to represent a primary dysregulation of gene expression in RAO, but rather occurs as a secondary effect following an abnormal response to allergens, e.g. monocyte/macrophage activation possibly driven by anti-apoptotic properties of IER3 or CDC20. Activated macrophages can be killed by the Vγ1 subset of γδ T cells in order to maintain homeostasis after infection. This process is mediated by a Fas-FasL interaction [100,101]. Delay in the apoptosis of neutrophils derived from bronchoalveolar lavage fluid (BALF) of the RAO horses has already been described [102]. Moreover, a significant decrease in the ratio of early apoptotic cells in the neutrophil population in the BALF of RAO horses has been recently shown [103]. The authors of these studies suggested a role for the granulocyte/macrophage colony stimulating factor (GM-CSF) in the delayed apoptosis. Our data possibly indicate that this decrease in the apoptosis rate might be additionally mediated by the higher expression of the anti-apoptotic *IER3* gene in RAO horses. The *IER3* gene is a known inhibitor of apoptosis induced by FAS or tumour necrosis factor (TNF)-α, and is regulated by NF-κB [104]. Schott et al. showed that IER3 protects murine macrophages from LPS-induced cell death [105]. However, *IER3* was upregulated in unstimulated RAO cells, whereas *CDC20* was significantly upregulated and involved in the most significant pathway upon all stimulations (Figs 3 and 5, S2 Table). *CDC20* is highly expressed in various types of human tumours [106,107] and studies have shown depleting endogenous CDC20 leads to induction of apoptosis [108,109]. The CDC20 binds to APC and promotes degradation of a proapoptotic protein, Bim [110].

The imbalance in macrophage homeostasis may trigger abnormal, allergic, immune responses. However, the role of *CDC20* in regards to macrophage homeostasis and RAO/asthma needs further investigation. Furthermore, clarifying the role of monocyte/macrophage activation in RAO may require investigations of these DE genes in the lung compartment of affected horses, since PBMC stimulation likely has limitations in this regard. In a murine model of asthma for instance, resident alveolar macrophages were shown to suppress inflammatory cytokine levels and eosinophil numbers. In contrast, recruited monocytes (which may correspond to the PBMC population) promoted allergic lung inflammation [111]. This suggests that future studies should investigate both the systemic (e. g. in PBMCs) and local (e. g. in resident macrophages and dendritic cells) responses in the RAO model of asthma.

### Future Perspectives

More than 15% of the DE genes identified in this study did not have an associated HGNC symbol, many horse genes have not been characterized yet, and most gene ontologies come from human, mouse or rat data. Thus, the pathway analysis of the equine data is incomplete and must be interpreted with caution.

However, the DE genes identified can serve as a valuable source of potential therapeutic targets in the treatment of equine RAO and human asthma. The HIF-1α subunit has been already proposed as a therapeutic target in asthma [112] and effectiveness of HIF-1 inhibitors in reducing the symptoms of allergic rhinitis has been reported in mouse models [113]. Also, *CXCL13*, which was significantly upregulated in RAO samples upon stimulation with LPS/HDE, has been recently proposed as a target gene in asthma treatment based on experiments performed in a mouse asthma model [114]. It also seems to play a key role in other immunologic diseases, e.g. autoimmune myasthenia gravis and multiple sclerosis [115,116]. Up to date, the role of *CXCL13* has not been investigated in well phenotyped asthmatic patients, and our study provides the first information on *CXCL13* expression differences in a natural model of asthma.

Interestingly, *CDC20* has not yet been considered as a putative therapeutic target in asthma. Studies with a CDC20 inhibitor may reveal more information on its role in RAO/asthma. *CDC20* may also be considered as a putative biomarker for RAO, since it was upregulated in RAO-samples upon each of the stimulations. In addition, studies on the function of *NDUFA4L* gene and its role in asthma could also reveal new insights in our understanding of alterations in the immune response or the tissue remodelling.

To further investigate the pathogenesis and mechanism of the immune response in RAO-affected horses and address the potential roles of the genes identified in the present study, global gene expression should be investigated in lung tissues of affected horses and controls, where the main immune response is taking place, at least for some of the DE genes identified in this study, such as the HIF-1 and NF-κB target genes.

However, the results for the stimulated PBMCs described here suggest that in addition to the local inflammation in the lung tissue, a systemic response (or at least a response from cells, which may have come from the lung, but are circulating in peripheral blood) plays a role [23,117–120]. This aspect should be further explored in PBMCs from RAO-affected horses during a natural allergen challenge provoking clinical exacerbation.

The results presented in this study also provide valuable information for future genetic studies aimed at finding expression quantitative trait loci (eQTLs) associated with the differentially expressed genes. These putative eQTLs could possibly contain causative variants influencing the host susceptibility to the disease and are potential genetic markers that might help breeders to reduce the occurrence of RAO.

## Conclusions

Our study revealed that a natural model of asthma, equine RAO, leads to differences in PBMC gene expression. Many of the DE genes are involved in cell cycle regulation, e.g. *CDC20*. *CXCL13* showed the strongest difference between RAO and control horses and might play an important role in RAO. This gene might be upregulated in monocytes/macrophages and/or the Th17 lymphocyte subset. Possible sources of this upregulation are: 1) increased ratio of activated to non-activated immune cells due to an increased expression level of apoptosis suppressor gene, *CDC20*; and/or 2) the hypoxic environment and upregulation of *HIF1A* in the lung.

## Supporting Information

S1 Fig Multidimensional scaling of the samples. All samples were plotted across three first principal components and coloured according to (A) stimulating factor, (B) sequencing flow cell, (C) horse ID number. Panels (D-J) show subsets of samples plotted across first two principal components: (D) samples stimulated with hay dust extract labelled by horse ID number; (E) unstimulated samples and samples stimulated with RCA_1; (F) unstimulated samples and samples stimulated with RCA_4; (G) samples stimulated with RCA_1 and RCA_4; (H) unstimulated samples and samples stimulated with RCA_1 or RCA_4; (I) unstimulated samples and samples stimulated with LPS; (J) unstimulated samples and samples stimulated with HDE_6, HDE_9, or HDE_12.
(PDF)

S2 Fig Family dependence of differentially expressed genes between RAO and control samples. For each of the two horse families and upon each stimulation (no stimulating factor (mock), lipopolysaccharides (LPS), recombinant cyathostomin antigen (RCA), and hay dust extract (HDE)) the tests for differential expression (DE) were performed. The bars represent the number of DE genes in each of the families that were significantly different between families indicating an influence of the genetic background of the horses (***Fisher’s exact test; p-value ≤ 0.001). Common DE genes identified in both families were coloured in yellow.
(PDF)

S3 Fig Legend for GeneGo pathway map. The legend explains all pathway objects and interaction used in the map in Fig 3.
(PDF)

S1 File R code used for the differential expression analysis. (PDF)

S1 Table Results of differential expression analysis–pairwise comparisons. Significantly (with false discovery rate FDR ≤ 0.05) differentially expressed genes between RAO and control horses for each stimulating factor and cohort are listed with log2 fold changes and FDRs.
(XLSX)

S2 Table Results of differential expression analysis–factorial effects. Genes significantly (false discovery rate FDR ≤ 0.05) regulated by overall RAO-effect with log2 fold changes for baseline RAO and every RAO-interaction effect studied.
(XLSX)

S3 Table Gene enrichment analysis with GeneGo and GeneCodis. For each stimulating factor a separate analysis was performed using the list of significant DE genes between RAO and control horses (FDR < 0.05). The file includes 5 sheets representing results for: 1) pathway maps; 2) GO processes; 3) process networks; 4) diseases (by biomarkers); and 5) KEGG pathways.
(XLSX)
