# Supplementary figures and images for: Impaired Cell Cycle Regulation in a Natural Equine Model of Asthma
Source: PLoS One. 2015 Aug 20;10(8):e0136103. doi: 10.1371/journal.pone.0136103 (PMC4546272; doi:10.1371/journal.pone.0136103)

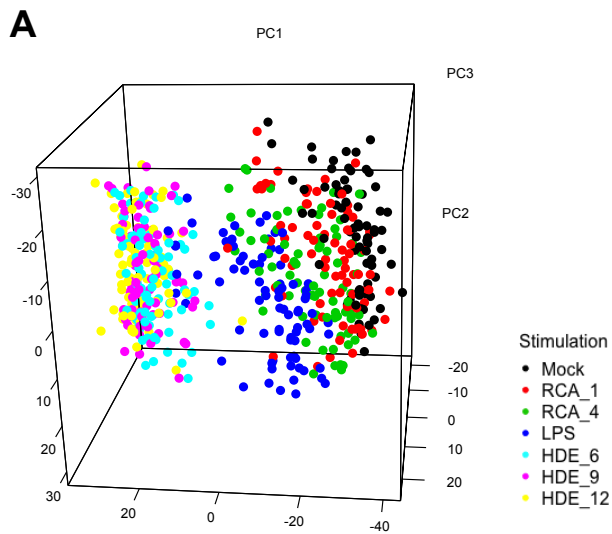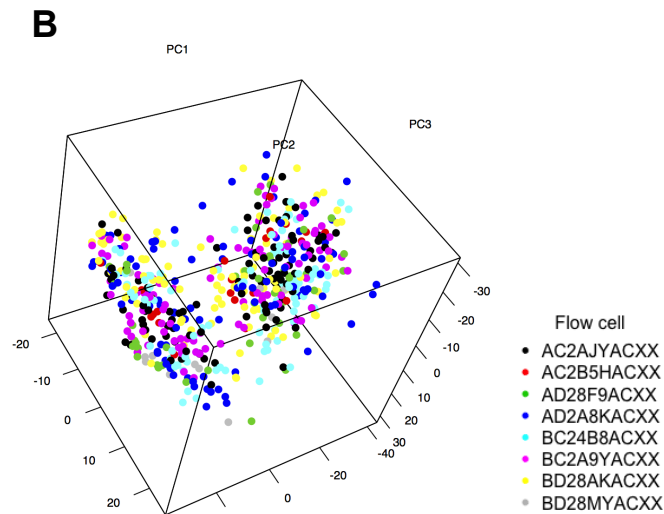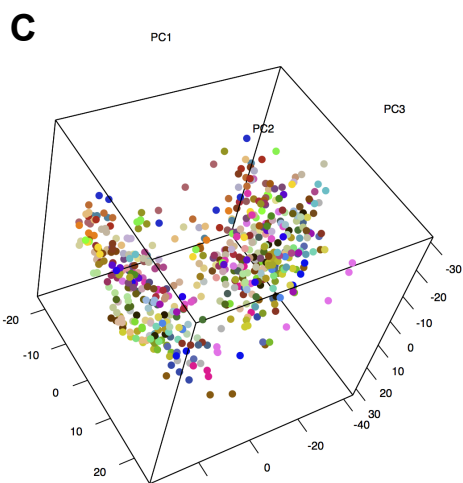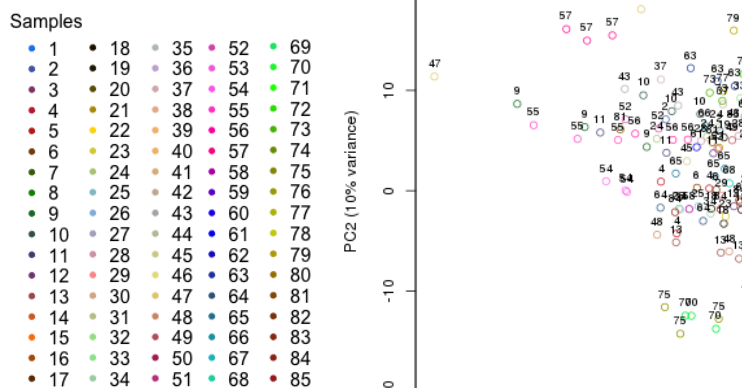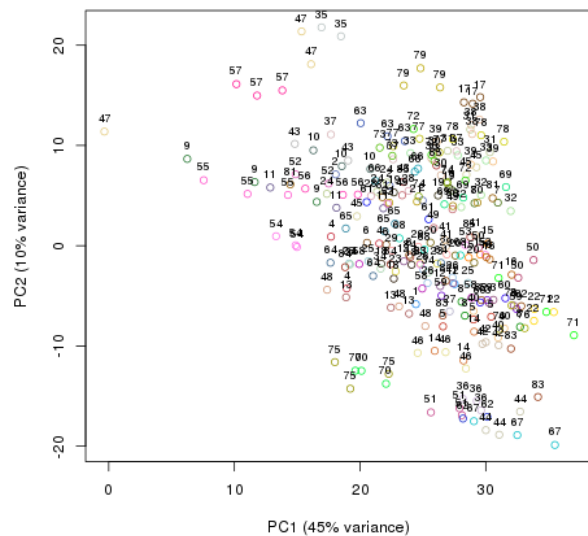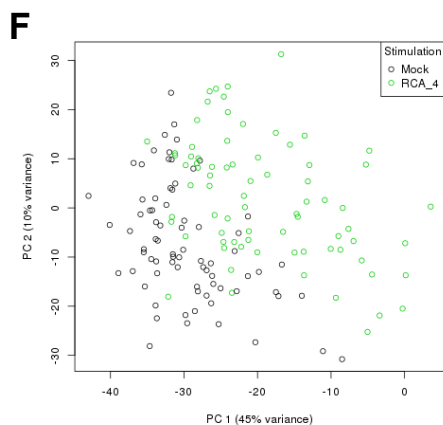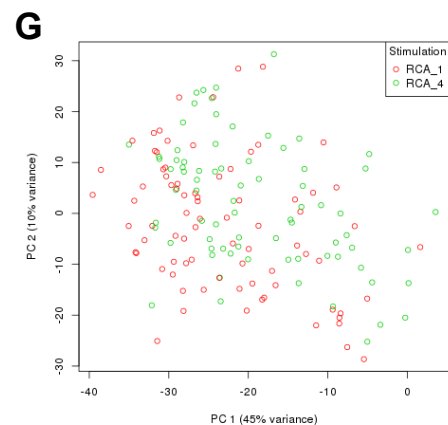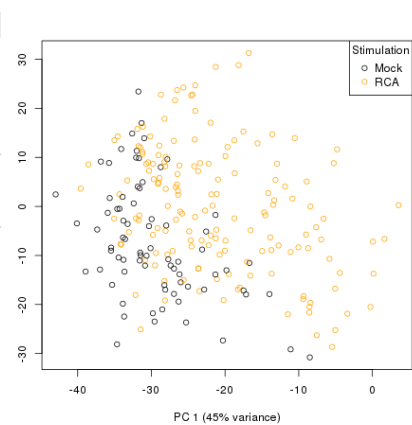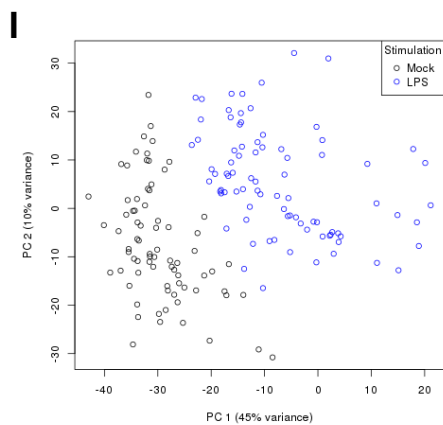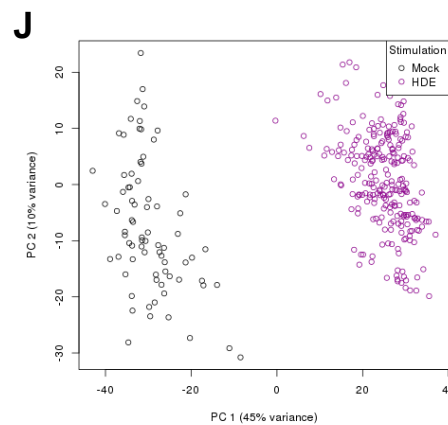

Supplement: S1 Fig — All samples were plotted across three first principal components and coloured according to (A) stimulating factor, (B) sequencing flow cell, (C) horse ID number. Panels (D-J) show subsets of samples plotted across first two principal components: (D) samples stimulated with hay dust extract labelled by horse ID number; (E) unstimulated samples and samples stimulated with RCA_1; (F) unstimulated samples and samples stimulated with RCA_4; (G) samples stimulated with RCA_1 and RCA_4; (H) unstimulated samples and samples stimulated with RCA_1 or RCA_4; (I) unstimulated samples and samples stimulated with LPS; (J) unstimulated samples and samples stimulated with HDE_6, HDE_9, or HDE_12. (PDF) [file pone.0136103.s001.pdf]

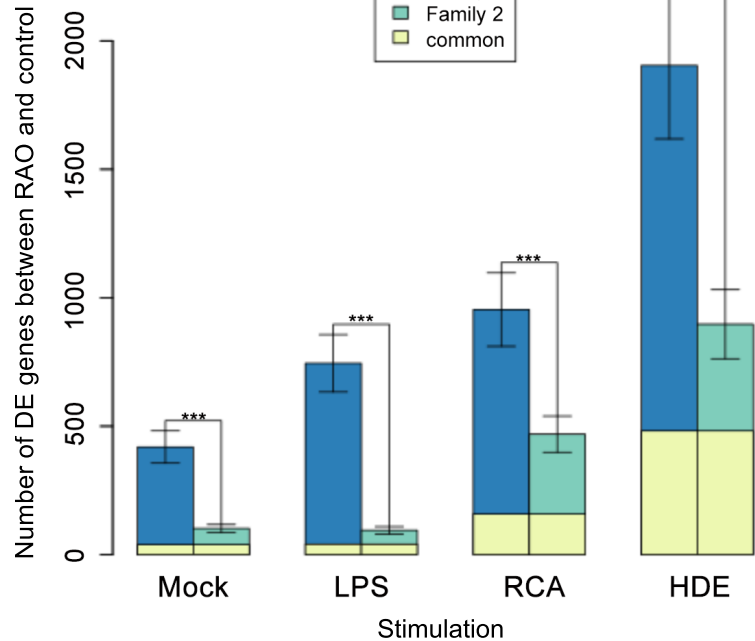

Supplement: S2 Fig — For each of the two horse families and upon each stimulation (no stimulating factor (mock), lipopolysaccharides (LPS), recombinant cyathostomin antigen (RCA), and hay dust extract (HDE)) the tests for differential expression (DE) were performed. The bars represent the number of DE genes in each of the families that were significantly different between families indicating an influence of the genetic background of the horses (***Fisher’s exact test; p-value ≤ 0.001). Common DE genes identified in both families were coloured in yellow. (PDF) [file pone.0136103.s002.pdf]
