# Supplementary material for: Impaired Cell Cycle Regulation in a Natural Equine Model of Asthma
Source: PLoS One. 2015 Aug 20;10(8):e0136103. doi: 10.1371/journal.pone.0136103 (PMC4546272; doi:10.1371/journal.pone.0136103)
Supplement: S3 Fig — The legend explains all pathway objects and interaction used in the map in Fig 3. (PDF) [file pone.0136103.s003.pdf]

METACORE QUICK REFERENCE GUIDE

USER DATA

NETWORKS / MAPS

- **Up-regulated (+)** — Object has user data with positive value
- **Down-regulated (-)** — Object has user data with negative value
- **Mixed-signal (+/-)** — Object has user data with both positive and negative values
- **Gene variants** — Object has user data with gene variants
- **Mixed data** — Object has user data with both expression values and gene variants

NETWORK OBJECTS

ENZYMES

- Generic enzyme

KINASE
- Generic kinase
- Protein kinase
- Lipid kinase

PHOSPHATASE
- Generic phosphatase
- Protein phosphatase
- Lipid phosphatase

PHOSPHOLIPASE
- Generic phospholipase

PROTEASE
- Generic protease
- Metalloprotease

GTPASE
- G-alpha
- RAS - superfamily

GENERIC CLASSES

- Receptor ligand
- Transcription factor
- Protein
- Compound
- Predicted metabolite or user's structure
- Inorganic ion
- Reaction
- DNA
- RNA
- Generic binding protein

CHANNELS/TRANSPORTERS

- Generic channel
- Ligand-gated ion channel
- Voltage-gated ion channel
- Transporter

RECEPTORS

- Generic
- GPCR
- Receptors with kinase activity

G PROTEIN ADAPTOR/REGULATORS

- G beta/gamma
- Regulators (GDI, GAP, GEF, etc.)

GROUPS OF OBJECTS

- **A complex or a group** — Proteins physically connected into a complex or related as a family
- **Logical association** — Proteins linked by logical relations or physical interactions
- **Custom association** (Group1) — Group of collapsed objects chosen by user

INTERACTIONS BETWEEN OBJECTS

EFFECTS

- Positive / activation
- Negative / inhibition
- Unspecified

MECHANISMS

PHYSICAL INTERACTIONS

- **B** — Binding: Physical interaction between molecules
- **C** — Cleavage: Cleavage of a protein at a specific site yielding distinctive peptide fragments. Proteolytic cleavage can be carried out by both enzymes and compounds
- **CM** — Covalent modifications: Covalent binding of a small chemical groups to protein amino acids or DNA/RNA nucleotides.
- **+P** — Phosphorylation: Protein activity is altered via addition of a phosphate group
- **- P** — Dephosphorylation: Protein activity is altered via removal of a phosphate group
- **T** — Transformation: Protein activity regulation by binding & hydrolysis of GTP
- **Tn** — Transport: Transport of a protein or a compound between organelles
- **Z** — Catalysis: Catalysis of an enzymatic reaction
- **Tr** — Transcription regulation: Physical binding of a transcription factor to target gene's promoter
- **cRT** — Co-regulation of transcription: Influences on gene expression by direct binding with transcription machinery or by chromatin remodelling
- **Rg** — Regulation: Influence on the biochemical reaction by changing its composition
- **M** — MicroRNA binding: Regulation of gene expression by binding of microRNA to target mRNA

FUNCTIONAL INTERACTIONS

- **IE** — Influence on expression: indirect influence of chemical compound or protein on the amount of another protein
- **Cn** — Competition: When two molecules compete for the interaction with the third molecule
- **?** — Unspecified interactions: Influence on activity of protein or RNA without determined mechanism
- **Pr** — Processing: Protein is a product of posttranslational modification.
- **PE** — Drug-Drug interactions. Pharmacological effect: Drugs change pharmacological effects of other drugs, for instance by competing for drug metabolism enzymes or organic transporters
- **TE** — Drug-Drug interactions. Toxic effect: Drugs change toxic effects of other drugs, for instance by competing for drug metabolism enzymes or organic transporters

LOGICAL RELATIONS

- **GR** — Group relation: Object belongs to a generic group of related objects
- **CS** — Complex subunit: Protein is a subunit of a protein complex
- **SR** — Similarity relation: Chemically similar compounds with chosen Tanimoto similarity score

LINKS ON NETWORKS

- **Incoming interaction** — When the mouse is over object, yellow link indicates direction to object
- **Outgoing interaction** — Cyan link indicates direction FROM the object

INTERACTIONS FROM CUSTOM LIST (MetaLink™)

- **Interaction is in the network** — Interaction is represented by a thin solid line and is highlighted in blue
- **Interaction is in the base, but not in network** — Interaction is highlighted in yellow
- **Interaction is in the network** — Interaction is highlighted in magenta

CANONICAL PATHWAYS

- **Canonical pathway** — The link is highlighted in a thick cyan or magenta line

LINKS ON MAPS

- Disrupts in disease
- Weakens in disease
- Emerges in disease
- Enhances in disease
- Species specific interactions

OBJECTS ON MAPS

LOCALIZATION

- Mitochondria
- EPR
- Golgi
- Nucleus
- Lysosome
- Peroxisome
- Cytoplasm
- Extracellular

OTHER MAP OBJECTS

Comments
- Note
- Normal process
- Pathological process

Blocks
- Normal process
- Pathological process

- Species specific object
- Path start

THOMSON REUTERS REGIONAL OFFICES

North America
Philadelphia
+1 800 336 4474
+1 215 386 0100

Latin America
+55 11 8370 9845

Europe, Middle East and Africa
Barcelona +34 93 459 2220
London +44 20 7433 4000

Asia Pacific
Singapore +65 6775 5088
Tokyo +81 3 5218 6500

Contact us to find out more about *MetaCore* or visit **thomsonreuters.com/diseaseinsight**

For a complete office list visit: **science.thomsonreuters.com/contact**

LS0312

Copyright © 2013 Thomson Reuters

THOMSON REUTERS™
