# Supplementary material for: Impaired Cell Cycle Regulation in a Natural Equine Model of Asthma
Source: PLoS One. 2015 Aug 20;10(8):e0136103. doi: 10.1371/journal.pone.0136103 (PMC4546272; doi:10.1371/journal.pone.0136103)
Supplement: S1 File — (PDF) [file pone.0136103.s004.pdf]

```
####################################################################
#------------------------------------------------------------------#
# Differential expression analysis of RNA-seq data
#------------------------------------------------------------------#
####################################################################
# Last modified: 05.05.2015
# Author: Alicja Pacholewska
# Article: Impaired Cell Cycle Regulation in a Natural Equine Model
# of Asthma. (PLOS ONE)
# Contact: alicja.pacholewska@vetsuisse.unibe.ch

# Binary sequence alignment/map (bam) files are available here:
# http://www.ebi.ac.uk/ena/data/view/PRJEB7497
# Annotations used for mapping and counting the mapped reads are
# available here:
# ftp://ftp.ensembl.org/pub/release-72/gtf/equus_caballus/
# Equus_caballus.EquCab2.72.gtf.gz

#------------------------------------------------------------------#
# FILES DESCRIPTION:
# RAOhorseRNAseq_DESIGN.txt - file with phenotypic data of the
#                              samples
# RAOhorseRNAseq_COUNTS.txt - file with tag counts per gene
#                              (genes from Ensembl release 72
# RAOhorseRNAseq_ANNOT.txt - file with annotations with following
# column names: Ensembl Gene ID,Gene symbol, Chromosome Name,
# Gene Start (bp),Gene End (bp),Description

# ABBREVIATIONS:
# DE = differentially expressed
# HDE = hay dust extract
# Mock = negative control (non-stimulated cell culture)
# LPS = lipopolysaccharides, "positive control" of the immune
response
#       (LPS also found in hay dust)
# RCA = recombinant cyathostomin antigen
# FDR = false discovery rate

#------------------------------------------------------------------#
# INSTALLING NECESSARY R PACKAGES
source("http://bioconductor.org/biocLite.R")
biocLite (pkgs=c("edgeR"))

#------------------------------------------------------------------#
#LOADING DATA FILES

counts<-
read.table('RAOhorseRNAseq_COUNTS.txt',header=TRUE,sep="\t",row.name
s=1)
# R by default adds "X" in front of numerical column names.
# This command is to remove "X" from the column names
colnames(counts)<-gsub("X","",colnames(counts))
design<-
read.table('RAOhorseRNAseq_DESIGN.txt',header=TRUE,row.names=1)
```

```
annot<-read.csv('RAOhorseRNAseq_ANNOT.csv',header=TRUE,row.names=1)

#prepare the data

# set "Mock" as a reference stimulus
design$stimulus<-factor(design
$stimulus,levels=c("Mock","LPS","HDE","RCA"))
# set "Un" as reference cohort
design$cohort<-factor(design$cohort,levels=c("Un","Fam1","Fam2"))
counts<-counts[,rownames(design)]
annot<-annot[rownames(counts),] # take annotations from analyzed
genes only
annot[,5]<-gsub(" \\[Source.*","",annot[,5])

#------------------------------------------------------------------#
# DIFFERENTIAL EXPRESSION ANALYSIS

#Load packages
library("edgeR")

#Create an object
group<-
factor(paste(design[,"condition"],design[,"stimulus"],design[,"cohor
t"],sep="_"))
dge<-DGEList(counts=counts,group=group,genes=annot)

#Keep only genes expressed >1cpm in at least mean number of samples
per group
keep <- rowSums(cpm(dge[,grep("Fam",design$cohort)])>1) >= 8 # keep
the genes expressed in families at least in 8 (mean number of
replicates) samples at level at least 1 count per million
dge<-dge[keep,]
keep <- rowSums(cpm(dge[,grep("Un",design$cohort)])>1) >= 25 # keep
the genes expressed in unrelated at least in 25 (mean number of
replicates) samples at level at least 1 count per million
dge<-dge[keep,]

#Normalize counts
dge<-calcNormFactors(dge)

#------------------------------------------------------------------#
# DESIGN MODEL - PAIRWISE COMPARISONS
#------------------------------------------------------------------#

#Using the GLM model approach we can investigate the effects of many
# factors simultaneously.
#We wanted to test the DE between RAO and CTL in every stimulation
# and every cohort. Therefore, we applied
# ~ 0 + group
# model that takes all three factors into account (condition,
# stimulation and cohort) and calculates coefficients for every
group.
# In total, we had 24 groups:
# [1] groupRAO_Mock_Fam1, groupRAO_Mock_Fam2, groupRAO_Mock_Un,
```

```
# [4] groupCTL_Mock_Fam1, groupCTL_Mock_Fam2, groupCTL_Mock_Un
# [7] groupRAO_LPS_Fam1,  groupRAO_LPS_Fam2,  groupRAO_LPS_Un,
# [10] groupCTL_LPS_Fam1,  groupCTL_LPS_Fam2,  groupCTL_LPS_Un
# [13] groupRAO_RCA_Fam1, groupRAO_RCA_Fam2,  groupRAO_RCA_Un,
# [16] groupCTL_RCA_Fam1,  groupCTL_RCA_Fam2,  groupCTL_RCA_Un
# [19] groupRAO_HDE_Fam1, groupRAO_HDE_Fam2,  groupRAO_HDE_Un,
# [22] groupCTL_HDE_Fam1,  groupCTL_HDE_Fam2,  groupCTL_HDE_Un

#Using the dummy coding we assigned values [0,1] to convey the group
# membership for each sample.
desPair<-model.matrix(~0+group,data=design)
colnames(desPair)

#Calculating coefficiency for every group (fitting the model):
dgePair <- estimateGLMCommonDisp(dge, desPair)
fitPair <- glmFit(dgePair,desPair)

#Make contrasts to compare RAO to control samples:
my.contrasts <- makeContrasts(
  #Condition and stimulation effects
  RAOvsCTL_Mock =
(groupRAO_Mock_Fam1/3+groupRAO_Mock_Fam2/3+groupRAO_Mock_Un/3)-
(groupCTL_Mock_Fam1/3+groupCTL_Mock_Fam2/3+groupCTL_Mock_Un/3),
  RAOvsCTL_LPS =
(groupRAO_LPS_Fam1/3+groupRAO_LPS_Fam2/3+groupRAO_LPS_Un/3)-
(groupCTL_LPS_Fam1/3+groupCTL_LPS_Fam2/3+groupCTL_LPS_Un/3),
  RAOvsCTL_HDE =
(groupRAO_HDE_Fam1/3+groupRAO_HDE_Fam2/3+groupRAO_HDE_Un/3)-
(groupCTL_HDE_Fam1/3+groupCTL_HDE_Fam2/3+groupCTL_HDE_Un/3),
  RAOvsCTL_RCA =
(groupRAO_RCA_Fam1/3+groupRAO_RCA_Fam2/3+groupRAO_RCA_Un/3)-
(groupCTL_RCA_Fam1/3+groupCTL_RCA_Fam2/3+groupCTL_RCA_Un/3),
  #Condition and stimulation effects in each cohort:
  RAOvsCTL_Mock_Fam1 = (groupRAO_Mock_Fam1)-(groupCTL_Mock_Fam1),
  RAOvsCTL_LPS_Fam1 = (groupRAO_LPS_Fam1)-(groupCTL_LPS_Fam1),
  RAOvsCTL_HDE_Fam1 = (groupRAO_HDE_Fam1)-(groupCTL_HDE_Fam1),
  RAOvsCTL_RCA_Fam1 = (groupRAO_RCA_Fam1)-(groupCTL_RCA_Fam1),
  RAOvsCTL_Mock_Fam2 = (groupRAO_Mock_Fam2)-(groupCTL_Mock_Fam2),
  RAOvsCTL_LPS_Fam2 = (groupRAO_LPS_Fam2)-(groupCTL_LPS_Fam2),
  RAOvsCTL_HDE_Fam2 = (groupRAO_HDE_Fam2)-(groupCTL_HDE_Fam2),
  RAOvsCTL_RCA_Fam2 = (groupRAO_RCA_Fam2)-(groupCTL_RCA_Fam2),
  RAOvsCTL_Mock_Un = (groupRAO_Mock_Un)-(groupCTL_Mock_Un),
  RAOvsCTL_LPS_Un = (groupRAO_LPS_Un)-(groupCTL_LPS_Un),
  RAOvsCTL_HDE_Un = (groupRAO_HDE_Un)-(groupCTL_HDE_Un),
  RAOvsCTL_RCA_Un = (groupRAO_RCA_Un)-(groupCTL_RCA_Un),
  levels=desPair
)

#Create object to store the results:
allGenesPair<-list()
deGenesPair<-list()

#DE tests
for (i in colnames(my.contrasts)){
```

```
  lrt<-glmLRT(fitPair, contr=my.contrasts[,i])
  effect<-topTags(lrt,n=length(rownames(lrt$table)))

#take only significantly DE genes with FDR threshold = 0.05
  x<-rownames(effect$table[effect$table$FDR<0.05,])
  deGenesPair[[i]]<-topTags(lrt,n=length(x))

write.table(deGenesPair[[i]],file=paste(i,"txt",sep="."),quote=FALSE
,sep="\t")
}

#Collect number of DE genes for each test performed
nb_deGenesPair<-list() #collect number of DE genes
for (i in names(deGenesPair)){
  nb_deGenesPair[i]<-length(rownames(deGenesPair[[i]]$table))
}

# Print matrix of numbers of DE genes for every comparison tested
# (contrast used)
as.matrix(nb_deGenesPair)

#Save results
save(dgePair,fitPair,allGenesPair,file="edgeRAOpair.RData")

#The list of differentially expressed genes can be shown using
# following command:
#   name="RAOvsCTL_HDE"
# DE genes between RAO samples stimulated with HDE compared
# to CTL samples stimulated with HDE
#   deGenesPair[[name]]

#The list of available tests (contrasts):
names(deGenesPair)


#------------------------------------------------------------------#
# DESIGN MODEL - FACTORIAL ANALYSIS
#------------------------------------------------------------------#

#We wanted to look for any RAO effect, regardless of
# stimulation/cohort. Therefore, we applied
# ~ condition * stimulus * cohort
# model that takes all three factors into account (condition,
# stimulation and cohort) and interactions between them.
# In total, we get 24 coefficients:

desFact<-model.matrix(~condition*stimulus*cohort,data=design)
colnames(desFact)

# [1] "(Intercept)”,     "conditionRAO”,  "stimulusHDE”,
# [4] ”stimulusLPS”,     "stimulusRCA”,   "cohortFam1”,
# [7] "cohortFam2”,      "conditionRAO:stimulusHDE”,
# [9] ”conditionRAO:stimulusLPS”, "conditionRAO:stimulusRCA”,
#[11] "conditionRAO:cohortFam1”,  "conditionRAO:cohortFam2”,
```

```
#[13] ”stimulusHDE:cohortFam1”,   "stimulusLPS:cohortFam1”,
#[15] ”stimulusRCA:cohortFam1”,   "stimulusHDE:cohortFam2”,
#[17] "stimulusLPS:cohortFam2”,   "stimulusRCA:cohortFam2”,
#[19] ”conditionRAO:stimulusHDE:cohortFam1”,
#[20] ”conditionRAO:stimulusLPS:cohortFam1”,
#[21] ”conditionRAO:stimulusRCA:cohortFam1”,
#[22] "conditionRAO:stimulusHDE:cohortFam2”,
#[23] ”conditionRAO:stimulusLPS:cohortFam2”,
#[24] "conditionRAO:stimulusRCA:cohortFam2"

#Calculating coefficiency for every factor
dgeFact<- estimateGLMCommonDisp(dge, desFact)
fitFact <- glmFit(dgeFact,desFact)

#overall RAO effect:
lrt <- glmLRT(fitFact, coef = grep("RAO",colnames(desFact)))
effect<-topTags(lrt,n=length(rownames(lrt$table)))
#filter DE genes with FDR < 0.05
x<-rownames(effect$table[effect$table$FDR<0.05,])
effect<-topTags(lrt,n=length(x))
#write results to an output text file
write.table(effect,file="overallRAOeffect.txt",quote=FALSE,sep="\t")


#Single RAO effects:
allGenesFact<-list()
deGenesFact<-list()

for (i in grep("RAO",colnames(desFact))){
 lrt <- glmLRT(fitFact, coef = i)
 effect<-topTags(lrt,n=length(rownames(lrt$table)))
 allGenesFact[[i]]<-effect
 #filter DE genes with FDR < 0.05
 x<-rownames(effect$table[effect$table$FDR<0.05,])
 deGenesFact[[i]]<-topTags(lrt,n=length(x))
 #write results to an output text file
 write.table(deGenesFact[[i]],file=paste(colnames(desFact)
[i],”txt",sep="."),quote=FALSE,sep="\t")
}


#Collect number of DE genes:
nb_deGenesFact<-list()
for (i in names(deGenesFact)){
 nb_deGenesFact[i]<-table(deGenesFact[[i]]$table$FDR<0.05)[2]
}
as.matrix(nb_deGenesFact)

names(allGenesFact)<-colnames(desFact)
save(fitFact,dgeFact,allGenesFact,file=“edgeRAOfact.RData”)

sessionInfo()
q()
```
